# Distinct Nutritional Profiles of Fermented *Chamerion angustifolium* from Natural, Organic, and Biodynamic Cultivation Systems: Evidence from a Four-Year Study

**DOI:** 10.3390/plants15071074

**Published:** 2026-04-01

**Authors:** Marius Lasinskas, Elvyra Jarienė, Jūratė Staveckienė, Jurgita Kulaitienė

**Affiliations:** 1 Department of Plant Biology and Food Sciences, Vytautas Magnus University Agriculture Academy, Donelaicio St. 58, 44248 Kaunas, Lithuania; elvyra.jariene@vdu.lt (E.J.); jurate.staveckiene@vdu.lt (J.S.); jurgita.kulaitiene@vdu.lt (J.K.); 2 Bioeconomy Research Institute, Vytautas Magnus University Agriculture Academy, Donelaicio St. 58, 44248 Kaunas, Lithuania

**Keywords:** cultivation systems, solid-state fermentation, functional foods, leaves

## Abstract

This four-year study assessed the nutritional profiles of fermented *Chamerion angustifolium* leaves from natural, organic, and biodynamic cultivation systems. Vitamins, pigments, and sugars were analyzed under standardized aerobic solid-state fermentation (SSF) for 24 and 48 h. Biodynamically grown leaves showed 42.7% and 49.7% higher β-carotene levels than organically and naturally grown leaves, while naturally grown leaves accumulated the most chlorophylls and total sugars. Organic cultivation promoted the highest vitamin C and L-ascorbic acid concentrations. Prolonged fermentation (48 h) significantly enhanced vitamin C (18.48%) and L-ascorbic acid (16.50%) only in naturally grown leaves. These findings demonstrate consistent cultivation-dependent nutritional trends and highlight the functional potential of fireweed leaves as bioactive ingredient carriers for functional foods, with applications in dairy products, beverages, and dietary supplements.

## 1. Introduction

Most studies on fermented fireweed (*Chamerion angustifolium* (L.) Holub) have focused on short-term effects or single-season comparisons, typically evaluating only phenolic compounds or antioxidant capacity. In contrast to earlier experiments, which primarily focused on biologically active substances in fresh naturally growing fireweed leaves, our study is the first to integrate four consecutive years of observations across natural, organic, and biodynamic farming systems, assessing how standardized aerobic solid-state fermentation (SSF) influences key nutritional attributes, such as vitamins, pigments, and sugars.

Researchers are increasingly exploring ways to use medicinal and food plants to support human health while preserving and cooperating with nature. Consequently, cultivation methods—including organic, biodynamic, and conventional systems—are being systematically evaluated for their ability to enhance the accumulation of biologically active phytochemicals [1]. After controlled cultivation and harvesting, appropriate post-harvest processing, including fermentation, is essential to optimize the extraction and bioavailability of these compounds. *Chamerion angustifolium* (fireweed) is rich in bioactive compounds, particularly carotenoids, polyphenols, tannins, chlorophylls, and vitamin C [2].

The bioactive compounds in fireweed enhance its antioxidant properties and potential health benefits, making it a valuable ingredient for functional foods [3,4,5].

Cultivating fireweed under organic or biodynamic systems can increase these compounds while minimizing environmental impact. Biodynamic farming is a form of improved organic farming, often certified by the Demeter system, that prohibits synthetic chemicals and uses special compost preparations, crop rotation, and biodiversity [6,7].

Solid-state fermentation (SSF), an eco-friendly technology, has been applied to improve the organoleptic and physicochemical properties of plant materials, enhancing the extraction of bioactive compounds [8,9,10]. It has attracted considerable interest in recent years, as it can be used for a variety of purposes (food, pharmaceutical, and biofuel industries) [11]. There are numerous advantages of solid-phase fermentation compared to fermentation in liquid media. Solid-state fermentation is particularly widely used to produce food products with improved biological value and safety, enriched with polyphenols and flavor compounds [12].

Solid-state fermentation is the process that takes place on a solid substrate, when there is no or almost no free water, but the substrate requires a small amount of moisture to support the growth and metabolic activity of microorganisms [13]. Solid-state fermentation does not use additional water, and the fermentation time is usually about 1–3 days, making this fermentation method more economically beneficial.

In addition, solid-state fermentation helps to avoid possible by-products of fungal metabolism, such as mycotoxins, resulting from their intensive and long-term activity in humid conditions, which are characteristic of conventional fermentation in a liquid medium. Solid-state fermentation has traditionally been used for the production of metabolites (enzymes, antibiotics, organic acids, aromatic compounds), but currently this fermentation method is gaining more and more attention due to its wider application for sustainable purposes when used for agricultural residues [14]. In this way, many alternative applications of solid-phase fermentation can be achieved, such as bioremediation, production of lipids, biofuels (biodiesel, bioethanol), flavors in the food industry, extraction of bioactive compounds, etc. [14].

One of the most popular fermented beverages in the world is tea. Fermented teas are obtained by biochemical processes in plant cells, as well as by the activity of microorganisms and enzymes. More biologically active substances are found in such teas, as new metabolites appear in them due to the activity of microorganisms and enzymes [15]. Teas prepared by this fermentation process are dominated by xerophytic fungi, mainly of the genus *Ascomycetes*: *Aspergillus*, *Penicillium*, *Saccharomyces*, and *Eurotium* [16].

Despite growing interest, the effectiveness of biodynamic and organic cultivation on the accumulation of bioactive compounds in fireweed leaves has not been thoroughly investigated. The present study aimed to evaluate the effects of cultivation systems and naturally aerobic SSF duration on pigments, vitamin C and derivatives, and sugars. The results provide practical guidance for functional food producers and farmers cultivating fireweed under sustainable systems.

## 2. Results and Discussion

### 2.1. The Amount of Pigments

In general, the growth and quality of agricultural products are strongly influenced by site conditions, with plant metabolism affected by soil quality and other environmental stressors. Abiotic stresses not only induce structural and anatomical changes in plants but also cause fluctuations in the concentrations of their chemical constituents.

The application of different cultivation and fertilization practices can either mitigate or exacerbate these stress factors [17,18].

In recent years, solid-state fermentation has emerged as a promising alternative to submerged fermentation due to its advantages, including reduced wastewater production, lower energy consumption, and higher productivity.

A statistically significant amount of β-carotene was found in biodynamically grown samples, i.e., 42.7% and 49.7% higher than in organic and natural leaves, respectively. A similar trend was observed in the content of lutein. In biodynamic leaves, its synthesis was more efficient and statistically significantly exceeded the levels in organic and natural leaves by 24.1% and 14.4%, respectively. The amount of zeaxanthin, regardless of the cultivation method, was similar across all samples. Only a slightly higher concentration was detected in leaves grown under natural conditions (Table 1). The naturally aerobic fermentation process had a significant influence on the carotenoid profile. The β-carotene content of all fireweed samples significantly decreased after fermentation, with the most notable content decrease of 49.7% observed in naturally grown leaves after 24 h. It was found that the zeaxanthin content remained stable throughout the entire technological process.

It is noteworthy that a slight decrease in lutein content was observed during fermentation in biodynamically grown leaves; however, a subsequent slight increase was recorded as the process progressed. It was observed that in organic and natural leaves, the fermentation process had the opposite effect on the content of lutein levels. In organic cultivated leaves, the content of lutein significantly increased by 23.7% after 24 h of fermentation, while in naturally grown leaves it increased by 12.9% after 48 h compared to the non-fermented samples. These findings suggest that both the cultivation method, particularly biodynamic and natural systems, and prolonged natural aerobic fermentation contributed to the modulation of lutein concentrations in fireweed leaves. A slightly lower amount of chlorophyll b was detected in organically grown samples, 5.7% and 16.9%, compared to the concentration found in the leaves from natural and biodynamic systems, respectively. Natural growth conditions were the most favorable for the synthesis of chlorophyll a in fireweed leaves.

A significantly higher concentration of chlorophyll a was observed under these conditions, 43.3% and 19.7% higher compared to biodynamic and organic leaves, respectively. Solid-state aerobic fermentation harmed both chlorophyll a and chlorophyll b levels across all cultivation systems (Table 1).

After 24 h of fermentation, the greatest decreases in chlorophyll b and a content were observed in naturally grown leaves, 34.7% and 61.1%, respectively.

Carotenoids and chlorophylls are widely present in plant products and play essential roles in human health [19]. The carotenoid lutein, in particular, confers multiple health benefits, including protective effects against neurological disorders, eye diseases, and skin conditions [20]. The pigment content in plants is regulated by the balance between their biosynthesis and degradation rates, as well as their stability within plastid storage structures [21,22]. Additionally, soil organic matter content may influence the epoxidation and de-epoxidation processes of carotenoids involved in the xanthophyll cycle [23].

The intensity of carotenoid synthesis in leaves depends more on light intensity, soil composition, and climatic conditions during the growing season than on the cultivation system alone. Our findings indicate that the concentrations of β-carotene and lutein in the leaves of biodynamically grown *Chamerion angustifolium* (fireweed) were significantly higher compared to those grown under organic or natural conditions. Biodynamic cultivation involves the application of specific preparations designed to enhance the biological activity of the soil, thereby improving nutrient availability and uptake throughout the growing season. According to Giannattasio et al. (2013) [24], the BD 500 preparation exhibits a mode of action comparable to that of the hormone auxin, promoting soil microbial activity.

The transformation of organic matter into more bioavailable nutrients for plants depends on soil microbial abundance and diversity. The results of the study by Spaccini et al. (2012) [25] showed that polysaccharides and compounds containing alkyl groups dominate the formulation and stimulate plant development. These authors also suggest that BD 501, which is considered an activated form of silica, may exert its effects at very low concentrations by enhancing the plant’s physiological responses.

According to Bacchus (2010) [26], BD 501 activates photosynthetic processes, thereby promoting overall plant development. Silicon-based preparations have also been reported to strengthen epidermal cell walls, increasing plant resistance to fungal pathogens and adverse environmental conditions, and are associated with more intensive synthesis of secondary metabolites, including β-carotene, compared to plants cultivated under natural or organic systems [27].

Al-Aghabary et al. (2005) [28] have suggested that silicon promotes the development of the plant system and increases the resistance of plants to the toxic effects of agrochemical compounds. Ma et al. (2007) [29] identified specific proteins involved in the active transport of silicon to stress-damaged tissues. In organic cultivation, the production of secondary metabolites and antioxidants, including lutein and beta-carotene, in plants can be stimulated by the limited availability of nitrogen due to environmental stress conditions during the growing season.

The application of composts derived from plant or animal origin, green manures, or microbial inoculants increases the activity of soil microbes, which stimulates the uptake of phosphorus and other trace elements essential for carotenoid synthesis. Improvements in soil ecological structure under organic systems can stimulate root development and nutrient acquisition, thereby contributing to increased concentrations of β-carotene in plant tissues. The solid-state fermentation process and enzymes produced during microbial metabolism, such as oxidases from lactic acid bacteria and yeast, degrade macromolecular compounds in fireweed leaves into lower molecular weight substances and secondary metabolites [30]. This process can lead to a reduction in the concentration of certain chemical constituents. The leaf maceration process facilitates the disruption of cell walls, thereby enhancing extraction efficiency and promoting the diffusion of bioactive compounds. This mechanism likely contributed to the observed increase in carotenoid concentrations following fermentation. Our results suggest that specific solid-state fermentation conditions may promote the accumulation of bioactive compounds in fireweed leaves [31,32].

The results show that the concentration of β-carotene decreased with increasing duration of aerobic fermentation, independent of the cultivation system. To date, no comparable studies have examined changes in β-carotene content in fireweed (*Chamerion angustifolium*) leaves during solid-state fermentation. However, research on other plants suggests that the decrease may be related to the metabolic activity of lactic acid bacteria. It has been shown that some species of *Lactobacillus* species have been reported to modulate enzymes involved in carotenoid biosynthesis or degradation. Some bacterial strains can synthesize antioxidant compounds or other metabolites that reduce the oxidative degradation of β-carotene. Cheng et al. (2023) [33] found that the initial decrease in β-carotene content in alfalfa due to oxidative processes increased in later stages of fermentation and may be related to changes in the composition of the microbial community.

We observed that SSF duration significantly increased the lutein content in the leaves of biodynamically and naturally grown fireweed (*Chamerion angustifolium*) leaves. This is likely related to biochemical changes that occur during fermentation, including the activity of microbes, especially lactic acid bacteria such as *Lactobacillus* spp., as well as enzymatic processes that may enhance lutein biosynthesis. The results of studies by Zuha, Vidhu (2022) [34], and Florence et al. (2023) [35] show a similar trend in lutein accumulation during fermentation. Therefore, to preserve or enhance the lutein content, it is necessary to consider the specific physiological and biochemical properties of the raw material and the technological parameters of the SSF process.

Organic fireweed leaves showed a slightly lower concentration of chlorophyll b, while leaves originating from natural systems had the highest content of chlorophyll a. Several factors may account for this outcome. Primarily, this can be attributed to the increased exposure to ultraviolet (UV) radiation in open environments. The concentration of leaf pigments may be higher if plants grow under natural conditions. This process is usually slower. Also, plants allocate resources for photosynthesis more effectively and synthesize a higher amount of chlorophyll if nitrogen is limited in the soil. Research shows that the application of organic composts promotes the accumulation of chlorophyll a and b in leaves due to better nutrient availability to plants and more favorable soil nitrogen dynamics [36].

The results showed that organic fertilizers significantly increased the amount of chlorophyll a in spinach (*Spinacia oleracea*) leaves and slightly increased the amount of chlorophyll b compared to plants fertilized with inorganic nitrogen compounds. In addition, it was found that the chlorophyll a (16.52 mg/L) and b (5.80 mg/L) content in the leaves of *Lepidium sativum* fertilized with compost was higher than in unfertilized control plants [37]. The biosynthesis of the photosynthetic pigments chlorophyll a and b may benefit from the use of particular preparations in biodynamic farming to increase soil microbial activity and enzyme function. This element has not been well researched, though. Our study’s results demonstrated that solid-state aerobic fermentation decreased the quantities of chlorophyll a and b in all cultivation systems, a trend that other researchers have previously noted. Degrain et al. (2020) [38] claim that the contents of chlorophyll a and b in African nightshade leaves drastically drop during fermentation when lactic acid is present. Ramírez et al. (2015) [39] stated that during fermentation, changes in pH can lead to the formation of new compounds such as Mg-free chlorophyll derivatives, a carotenoid with 5,8-epoxide groups, and brown pigments (0-quinones). As a result of the oxidation reaction, the intensity of the leaves’ green color changes, leading to a decrease in chlorophyll content.

### 2.2. The Amount of Sugar

Our findings showed that the growing systems had a significant effect on the content of sugars and their derivatives. Naturally grown fireweed leaves exhibited significantly higher concentrations of total sugars and fructose, whereas the lowest sucrose concentration was observed in biodynamically grown leaves, and the lowest glucose concentration in organically grown leaves (Table 2).

Solid-state fermentation for 24 and 48 h did not significantly influence the total sugar content in leaves grown under biodynamic and organic systems. In contrast, naturally grown leaves exhibited a 12.13% reduction in total sugar content after 48 h of aerobic solid-state fermentation compared to unfermented leaves. However, an opposing trend was observed for sucrose content, which increased following fermentation, independent of the cultivation system. It should be noted that after 48 h of fermentation, the increase in naturally grown leaves was the most pronounced, 85.5% compared to control samples. The results of the study showed that during the fermentation process, the glucose content decreased regardless of the growing system, with the most significant reductions observed in biodynamically and naturally grown leaves, by 16% and 28.6%, respectively. The fermentation process had no significant effect on fructose content and varied only slightly across all analyzed samples (Table 2).

Sugar is a primary source of energy, and its accumulation in plants is influenced by various factors [40]. When grown organically, nitrogen is gradually released in the compost, allowing slower, more uniform growth and a more intense accumulation of sugars that are not used for vegetative development. It is likely that specific preparations (compost extract, silica solution, etc.) used in biodynamic farming help to balance the development of the plant. In both farming methods, the use of such preparations improves the soil microbiota, activates enzyme activity, and thus may increase the availability of nutrients, enhance the process of photosynthesis, and stimulate sugar biosynthesis in the tissues. This is also supported by the results obtained in our research: a slightly higher glucose content was found in biodynamically grown leaves. However, the results of the studies carried out show an ambiguous effect of cultivation technologies on sugar accumulation and carbohydrate composition in different plants.

Conti et al. [41] and Cayuela et al. [42], while studying the effect of different cultivation technologies on the quality of strawberries, found that in organically grown berries the sugar concentration was significantly higher. However, the data from other researchers did not confirm this finding, as very minor differences were observed [43]. Research by Mihálka et al. [44] showed that organically grown berries contained higher amounts of glucose and fructose compared to conventionally grown. The experimental results by Bavec et al. [45] demonstrated that biodynamic cultivation of red beet was more effective as it led to a higher accumulation of sugar content in the tissue compared to beets grown under an intensive farming system.

During solid-phase fermentation, hydrolytic processes break down complex substrates into simpler compounds. As a result, a substantial reduction in total sugar content was observed during aerobic solid-state fermentation, accompanied by a transient increase in glucose and fructose concentrations. This decrease in the amount of total sugars can be attributed to the enzymatic hydrolysis of the disaccharide sucrose into monosaccharides, a process likely mediated by microorganisms naturally present on the leaf surface.

Likely, the naturally occurring microbiota on the fireweed (*Chamerion angustifolium* (L.) Holub) leaves is dominated by the lactic acid bacteria, particularly species of *Lactobacillus*, *Leuconostoc,* and *Pediococcus*. During aerobic fermentation, these bacteria convert available sugars into lactic acid, resulting in a more acidic environment in the surrounding medium. During fermentation, actively participating yeasts such as *Saccharomyces* and *Candida* are capable of metabolizing sugar into ethanol, which can subsequently be oxidized into acetic acid or other compounds by acetic acid bacteria. For yeasts and lactic acid bacteria, the main energy sources are fructose and glucose, which the latter being consumed more rapidly, resulting in a noticeable decrease in its concentration. In our experiment, higher concentrations of fructose and glucose were detected in non-fermented (control) fireweed leaves.

In the Methodological part, we mentioned that the leaves were not additionally treated, so the natural epiphytic microbiota present on the leaf surface was not completely eliminated. Such microorganisms may contribute to biochemical changes during solid-state fermentation. The observed decrease in glucose content may indeed be associated with microbial metabolism, as glucose is one of the most readily utilized saccharides.

### 2.3. The Amount of Vitamin C and Its Derivatives

Organically grown fireweed leaves exhibited the highest statistically significant concentrations of vitamin C and L-ascorbic acid, i.e., 9.29% and 11.34%, higher, compared to the biodynamically grown ones, respectively. In contrast, the lowest content of both was observed in naturally grown fireweed leaves.

A short-term fermentation process (24 h) enhanced vitamin C and L-ascorbic acid levels across all cultivation systems, with the most pronounced increase detected in leaves grown under biodynamic and organic systems. However, a prolonged fermentation period of 48 h resulted in a decline in vitamin C and L-ascorbic acid concentrations. Notably, fermentation appeared more effective in naturally grown leaves, which demonstrated a relatively greater increase in vitamin C (18.48%) and L-ascorbic acid (16.50%) contents compared to their unfermented counterparts. The cultivation method and fermentation duration did not significantly affect the content of dehydroascorbic acid. A longer fermentation duration process (48 h) slightly increased its content, especially in organic leaves: 16.50% compared to unfermented ones (Table 3). During fermentation, the leaves are crushed and pressed, which enhances the degradation processes of the cell walls and improves the diffusion of biologically active compounds from the inner parts of the cells. This results in a more efficient process of extracting compounds. On the other hand, the results can be explained by the fact that vitamin C is very sensitive to atmospheric conditions (heat, light, air); therefore, it can be destroyed during plant raw material processing. There are some data suggesting that certain solid-phase fermentation parameters can activate the accumulation process of certain bioactive compounds in fireweed leaves [46,47].

Differences in ascorbic acid content in fireweed leaves may result from variations in cultivation systems and parameters of the natural fermentation process. We have already discussed that one of the key aims of organic and biodynamic cultivation is to enhance the diversity and vitality of soil microbiota, which in turn improves nutrient availability and supports overall plant health. This is also confirmed by our research results, which showed that the fireweed leaves grown biodynamically and organically contain slightly higher vitamin C than those naturally grown. In a study by Serri et al. [48] comparing the effect of different fertilization backgrounds on coriander (Coriandrum sativum), found that under the application of amino-acid glycine treatment, significantly increase catalase enzyme activity (12%), root biomass (36%), and vitamin C content (40%) compared to control plants.

In organic and biodynamic farming systems, plants are more frequently exposed to natural environmental conditions, which can induce mild oxidative stress. This physiological stimulus activates defense responses, including the increased biosynthesis of some antioxidant compounds, such as vitamin C, which are integral to the plant’s protective mechanisms. Supporting this, findings by Oliveira et al. [49] demonstrated that organically grown tomatoes, subjected to higher oxidative stress, accumulated 55% more vitamin C compared to those cultivated under intensive technology.

Koh et al. [50] in a comparative study on the quality of spinach grown under different technologies, also confirmed the above-mentioned statement that organic leaves contained lower levels of nitrates but exhibited higher concentrations of flavonoids and ascorbic acid compared to those cultivated under intensive technology. However, contrasting findings have also been reported. Ponder and Hallmann’s [51] studies results indicate that conventionally grown raspberries accumulated significantly higher levels of vitamin C compared to organic grown raspberries.

The activity of microorganisms present on fireweed leaves during natural aerobic solid-phase fermentation may account for the observed changes in vitamin C and its derivatives. During the preparation of the plant raw material for solid-phase fermentation, the leaves of the fireweed are mechanically shredded, leading to the disruption of cell walls. This process may facilitate the release of vitamin C, but at the same time it simultaneously increases exposure to oxidative effect which can intensify the degradation of its.

Vitamin C content is generally unstable and influenced by multiple factors. Yuan and Chen [52] proposed that ascorbic acid degradation occurs via two pathways: an oxidoreductive pathway involving the formation of dehydroascorbic acid (DHAA), and a hydrolytic pathway characterized by direct cleavage of the lactone ring of the ascorbic acid molecule. A similar trend in research results was observed in this experiment as well. Previous experimental results from our studies showed that the content of vitamin C in fireweed leaves decreased by 37% and 78%, after 24 and 72 h of aerobic fermentation, respectively, compared to levels in the unfermented leaves [53].

Changes in leaf mass after solid-state fermentation were not directly evaluated in this study. Potential variations in leaf mass may influence the interpretation of metabolite concentrations and should be considered in future studies. All samples were processed and analyzed under identical experimental conditions; therefore, relative comparisons between the studied groups remain valid.

### 2.4. Pearson’s Correlation

Table 4 shows Pearson’s correlation coefficients (r) between cultivation system (biodynamic, organic, and natural), fermentation durations (0 h, 24 h, and 48 h), and pigment concentrations in fireweed (*Chamerion angustifolium* (L.) Holub) leaves. Clear differences were observed: shorter fermentation promoted β-carotene accumulation, as indicated by moderately significant correlations between β-carotene and both lutein (r = 0.389) and biodynamic 24 h samples (r = 0.488). Conversely, longer 48 h fermentation in the organic samples showed a negative correlation with β-carotene (r = −0.434), suggesting pigment degradation over time.

Chlorophyll a (r = 0.716) and chlorophyll b (r = 0.598) exhibited strong positive correlations with non-fermented samples.

Table 5 summarizes Pearson’s correlation coefficients (r) between sugar content and cultivation system, fermentation duration in fireweed (*Chamerion angustifolium* (L.) Holub) leaves. In biodynamically grown leaves, glucose content showed a moderately positive correlation with non-fermented samples (biodynamic—0 h; r = 0.576), suggesting that fermentation reduces the amount of free glucose. In contrast, sucrose displayed a negative correlation with biodynamic non-fermented samples (biodynamic—0 h; r = −0.529), indicating that it may undergo hydrolysis into reducing sugars during fermentation.

In organically grown leaves, total sugar content (r = 0.734) and fructose (r = 0.545) were strongly positively correlated with non-fermented samples. However, after 48 h of fermentation, glucose showed a negative correlation (r = −0.521), while sucrose was positively correlated (r = 0.578). These results suggest that prolonged fermentation either decreases glucose availability or promotes the reconversion of simple sugars into disaccharides.

In naturally grown leaves, non-fermented samples exhibited negative correlations with fructose (r = −0.433) and total sugar (r = −0.258), whereas 24 h fermentation showed a positive correlation with sucrose (r = 0.487). The 48 h fermentation period demonstrated a negative association with total sugar (r = −0.454), indicating that extended fermentation leads to gradual sugar degradation. Overall, the correlation patterns show that fermentation modifies sugar balance in different ways depending on the cultivation system: longer fermentation, particularly under organic conditions, favored sucrose accumulation and decreased sugar depletion, while biodynamic and natural leaves retained higher glucose and total sugar levels in early stages.

Table 6 shows Pearson’s correlation coefficients (r) between vitamin C components and cultivation system and fermentation duration in fireweed (*Chamerion angustifolium* (L.) Holub) leaves. In biodynamically grown leaves, weak to moderate correlations were found.

Under natural cultivation, 48 h of fermentation showed positive correlations with total vitamin C (r = 0.418). These results indicate that prolonged fermentation in naturally grown leaves may enhance vitamin C redox cycling, thereby preserving the overall vitamin C content. In contrast, non-fermented samples exhibited negative correlations across all vitamin C forms (r = −0.260 to −0.404), consistent with oxidative losses during post-harvest handling.

In organic cultivation, 24 h fermentation demonstrated strong positive correlations with both total vitamin C (r = 0.634) and L-ascorbic acid (r = 0.631). However, after 48 h, total vitamin C (r = −0.273) and L-ascorbic acid (r = −0.614) showed negative correlations, while dehydroascorbic acid displayed a positive correlation (r = 0.568), suggesting progressive oxidation of the reduced form into its oxidized counterpart.

Among the vitamin C components, total vitamin C and L-ascorbic acid were highly positively correlated (r = 0.850), indicating that total vitamin C levels are predominantly determined by the reduced form. Overall, the correlation matrix reveals that both fermentation duration and cultivation method significantly influence vitamin C stability and interconversion between its reduced and oxidized states. While prolonged fermentation (48 h) tended to promote oxidation, particularly in organically grown samples, shorter fermentation (24 h) generally favored the preservation of ascorbate.

## 3. Materials and Methods

### 3.1. Methodology of the Experiment

**Figure 1:**
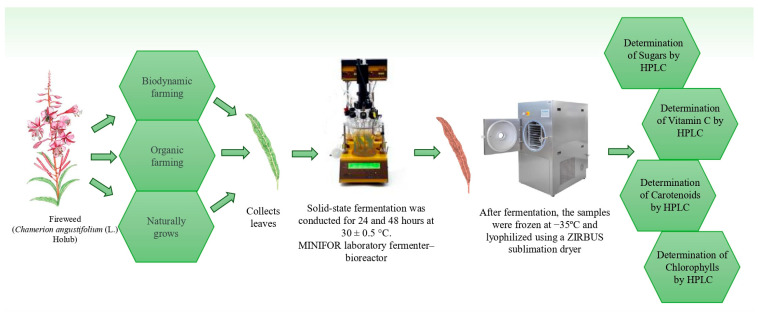
Methodology of the experiment.

The methodological table summarizes the experimental variants and the main stages of the study, including fermentation conditions, sample preparation, and analytical methods applied (Figure 1. Methodology of the experiment.).

### 3.2. Field Experiment

The field experiment was conducted at Giedre Naceviciene’s organic farm (certificate No. SER-T-19-00910), Safarkos village, Jonava district, Lithuania (55°00′22″ N, 24°12′22″ E). The soil in the experimental field was slightly acidic, with high phosphorus content, low calcium content, high humus content, and very low mineral nitrogen content. Meteorological conditions during the growing seasons (April–July) of 2021–2024 were comparable to long-term averages, except for the unusually warm summer of 2021 and slightly above-average conditions in 2024. Interannual variability was therefore considered minor, and multi-year averages were used for data interpretation.

**Figure 2:**
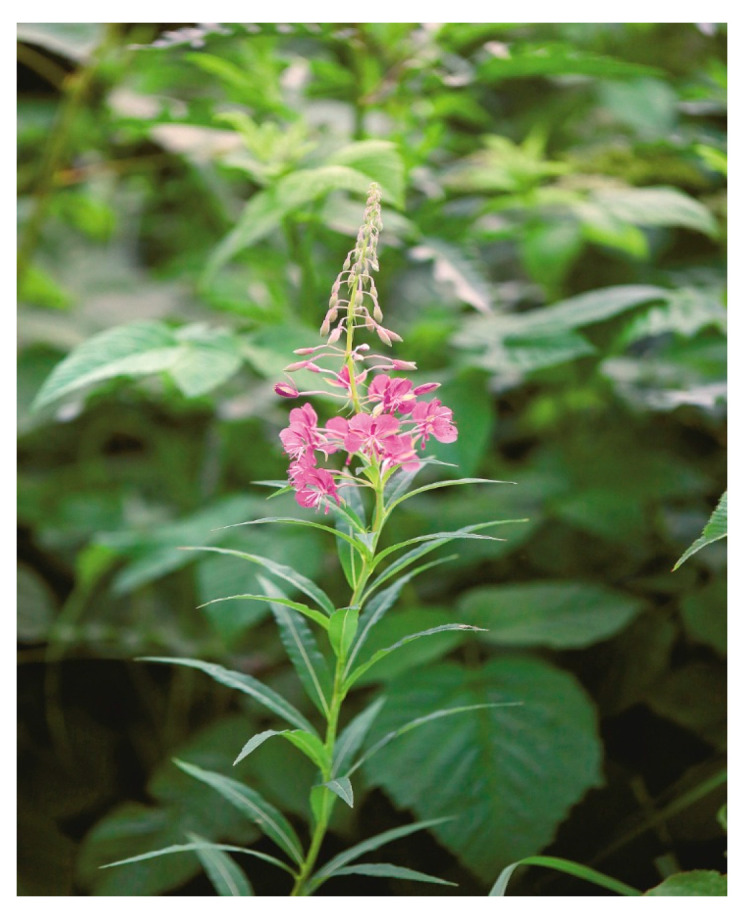
Fireweed plant (photo by author).

Fireweed (*Chamerion angustifolium* (L.) Holub) plants (Figure 2) were cultivated under organic and biodynamic farming systems and compared with naturally occurring wild populations, which served as the control. Plants were cultivated in rows with 70 cm spacing between rows and 30 cm between plants within rows. Approximately two weeks before the start of vegetation (mid-June), soil between rows was fertilized with either organic compost (25 t ha^−1^; mineral N: 52.7 mg kg^−1^, available P: 1932 mg kg^−1^, pHKCl: 6.97) or biodynamic compost (25 t ha^−1^; mineral N: 51.1 mg kg^−1^, available P: 1591 mg kg^−1^, pHKCl: 6.83).

### 3.3. Cultivation Treatments

#### 3.3.1. Natural Growing

The natural growing fireweed leaves were randomly collected from three replications (field plots) at the beginning of mass flowering (1 July decade). Leaves were harvested around noon when they were dry.

#### 3.3.2. Organic Treatment

Soil was sprayed with water in mid-May. Fireweed leaves were sprayed with water twice per growing season: (a) at the leaf formation stage (mid-June) and (b) at the beginning of mass flowering (early July).

#### 3.3.3. Biodynamic Treatment

Soil application: BD preparation 500 (fermented cow manure; total N: 2.28%, available K: 291 mg kg^−1^, available P: 1668 mg kg^−1^, pHKCl: 6.86, saccharase activity: 31.8 mg glucose g^−1^ soil 48 h^−1^, urease activity: 1.64 mg NH_3_ g^−1^ soil 24 h^−1^) was applied as a 1% solution (200 L ha^−1^) in mid-May.

Leaves were sprayed twice during the vegetation period with BD preparation 501 (0.5% solution, 200 L ha^−1^): (a) at leaf formation (mid-June) and (b) at the onset of mass flowering (early July). BD 501 consisted of finely ground quartz (SiO_2_: 99.8%). All biodynamic treatments followed standard European biodynamic practices. No pesticides or fungicides were applied. All biodynamic materials were obtained from a Demeter-certified supplier (CvW KG, Internationale Biodynamische Präparatezentrale, Künzelsau-Mäusdorf, Germany).

### 3.4. Preparation of the Samples

The fireweed leaves were randomly collected from three replications (field plots) in each farming system at the beginning of mass flowering (1 July decade). For each cultivation variant, three field plots were selected for leaf collection.

The total sample of the leaves was 10.8 kg/per farming system:

- Naturally grown: 3.6 kg (not fermented) for control, 3.6 kg for solid-phase fermentation lasting 24 h, and 3.6 kg for 48 h fermentation;
- Organically grown fireweed: 3.6 kg (not fermented) for control, 3.6 kg for solid-phase fermentation lasting 24 h, and 3.6 kg for 48 h fermentation;
- Biodynamically grown fireweed: 3.6 kg (not fermented) for control, 3.6 kg for solid-phase fermentation lasting 24 h, and 3.6 kg for 48 h fermentation;
- Control (0 h)—not fermented leaves, but were stored for the intended time;
- Three subsamples (1.200 kg for each variant) were prepared from the raw material.

### 3.5. Solid-State Fermentation

**Figure 3:**
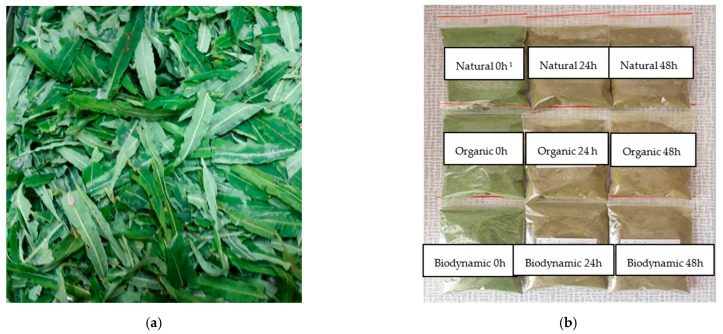
Not fermented (control) (**a**) and lyophilized fireweed leaves (powders) (**b**) ^1^ 0 h, 24 h, and 48 h—fermentation duration.

The collected fresh leaf samples were carefully rinsed with distilled water in order to minimize surface contamination by bacteria and fungi that could potentially influence the fermentation process; no sterilization or antimicrobial treatment was applied. The chopped fresh fireweed (*Chamerion angustifolium*) leaves (Figure 3a), prepared using a Thermomix TM-5 (Vorwerk, Wuppertal, Germany), were placed into a 1 L glass fermentation vessel covered with a lid allowing oxygen circulation and integrated into a MINIFOR (KIT) laboratory fermenter-bioreactor (LAMBDA Instruments GmbH, Baar, Switzerland). Solid-state fermentation was conducted for 24 and 48 h at 30 ± 0.5 °C (Figure 3b). After fermentation, the samples were frozen at −35 °C and lyophilized using a ZIRBUS sublimation dryer (ZIRBUS Technology, Bad Grund, Germany). The lyophilized leaves were then ground into a fine powder using a Grindomix GM 200 laboratory mill (Retsch GmbH, Haan, Germany) for further analyses.

### 3.6. Analytical Methods

#### 3.6.1. Determination of Sugars

A 100 mg fireweed sample was vortexed with 5 mL of 80% acetone. Extraction was carried out in an ultrasonic bath (5.5 kHz, 30 °C, 10 min). The mixture was centrifuged at 6000 rpm (3 °C, 10 min), and 1000 µL of the supernatant was transferred into HPLC vials. Sugar analysis was performed using a Shimadzu HPLC system equipped with two LC-20AD pumps, a CBM-20A controller, a SIL-20AC column oven, and a refractive index detector (RID) (Shimadzu USA Manufacturing Inc., Canby, OR, USA). Sugars were separated isocratically with 80% acetone in deionized water as the mobile phase (flow rate: 1 mL min^−1^). The total time was 15 min. A Phenomenex Luna NH_2_ column (Phenomenex, Torrance, CA, USA) was used. Glucose, fructose, and sucrose were identified and quantified against certified standards (≥99.9% purity, Sigma-Aldrich, Poznań, Poland) [54].

#### 3.6.2. Determination of Vitamin C

Vitamin C content was determined by HPLC coupled with a UV–VIS spectrophotometer (Shimadzu Manufacturing Inc., Canby, OR, USA) [54]. Approximately 100 mg of freeze-dried powder was extracted with 5 mL of 5% metaphosphoric acid (10 min, 30 °C, 5.5 kHz ultrasonic bath). The extract was centrifuged at 6000 rpm (0 °C, 10 min). A 100 µL aliquot was transferred into HPLC vials. The mobile phase consisted of an acetic acid buffer (pH 4.4) prepared from 0.1 M sodium acetate and 0.1 M glacial acetic acid (99.9% purity) in a 63:37 (*v*/*v*) ratio. Separation was performed isocratically at 1 mL min^−1^.

#### 3.6.3. Determination of Carotenoids

5 mL of acetone was added to 100 mg of freeze-dried fireweed powder. After ultrasonication (15 min, 0 °C), the extract was centrifuged at 6000 rpm (10 min, 0 °C). The supernatant was collected into HPLC vials. Separation was performed on a 250 × 4.6 mm Max-RP 80A column with an injection volume of 100 µL. Detection was carried out at 445–450 nm for 18 min. Quantification of carotenoids was achieved using external standards (≥99.9% purity, Sigma-Aldrich, Poland) [54].

#### 3.6.4. Determination of Chlorophylls

Freeze-dried samples (100 mg) were extracted with 100% acetone in an ultrasonic bath (10 min, 0 °C, 5.5 kHz). Extracts were centrifuged at 6000 rpm (0 °C, 10 min), and 1 mL of the supernatant was transferred into HPLC vials (Shimpol, Warsaw, Poland). Detection was performed at 445–450 nm. Chlorophyll content was expressed as mg per 100 g dry matter [54].

### 3.7. Statistical Analysis

All experiments and chemical analyses were carried out in triplicate. Data were analyzed using Statgraphics Centurion 15.2.11.0 (StatPoint Technologies, Inc., Warrenton, VA, USA). A two-way analysis of variance (ANOVA) was applied to evaluate the effects of farming system, fermentation duration, and their interaction on the measured parameters. Results are presented as least-squares means (LS means) ± SE (standard error) derived from the ANOVA model. When significant effects were observed (*p* < 0.05), Tukey’s post hoc test was used for multiple comparisons between groups. Pearson’s correlation coefficient was calculated using XLSTAT (Addinsoft, New York, NY, USA, 2018) to assess relationships among carotenoids, chlorophylls, vitamin C, and sugars in fireweed samples.

## 4. Conclusions

This study provides novel insights into the functional potential of fireweed leaves, showing that cultivation method and fermentation process significantly affect pigments, vitamin C, and sugar content.

Biodynamic cultivation favored the accumulation of carotenoids, particularly β-carotene and lutein, while zeaxanthin remained stable across all systems and processing stages.

Organic cultivation led to the highest concentrations of vitamin C and L-ascorbic acid, and short-term fermentation further enhanced their levels.

Naturally grown leaves accumulated more total sugars and fructose, whereas sucrose content increased during fermentation regardless of the cultivation system. Solid-state aerobic fermentation reduced chlorophyll a and b contents in all samples.

Solid-state fermentation also modulates nutritional value: increases some biologically active substances, such as tannin oenothein B and ellagic acid (which we observed in our previous experiments), but decreases the amounts of chlorophylls; vitamin C and sugar quantities were different according to cultivation methods and fermentation duration. Therefore, further research will be conducted to determine which fermentation parameters are most suitable.

Overall, the findings highlight fireweed leaves as a promising source of bioactive compounds for the development of functional foods, particularly for incorporation into dairy products, beverages, and dietary supplements.

## Figures and Tables

**Table 1:** The content of carotenoids and chlorophylls in fireweed (*Chamerion angustifolium* (L.) Holub) leaves (in mg 100 g^−1^ D.M.).

Variable	ß-Carotene	Zeaxanthin	Lutein	Chlorophyll b	Chlorophyll a
Natural 0 h	9.23 ± 0.44 ^1^ c ^2^	15.14 ± 0.02 a	27.90 ± 1.79 b	211.56 ± 0.93 a	358.46 ± 3.01 a
Natural 24 h	4.64 ± 0.11 d	15.09 ± 0.01 a	28.96 ± 1.92 b	138.06 ± 3.28 b	139.57 ± 7.44 d
Natural 48 h	4.93 ± 0.25 d	15.12 ± 0.01 a	31.51 ± 1.87 a	107.54 ± 2.80 c	86.15 ± 2.86 e
Biodynamic 0 h	18.33 ± 0.150 a	15.08 ± 0.01 a	32.60 ± 2.30 a	191.29 ± 6.22 a	203.17 ± 2.43 c
Biodynamic 24 h	17.65 ± 0.38 a	15.10 ± 0.01 a	29.86 ± 1.59 ab	145.00 ± 3.22 b	92.65 ± 1.39 e
Biodynamic 48 h	15.17 ± 0.31 b	15.10 ± 0.01 a	36.89 ± 0.73 a	119.70 ± 2.57 c	71.57 ± 0.44 f
Organic 0 h	10.50 ± 0.17 c	15.10 ± 0.01 a	24.75 ± 1.52 c	180.96 ± 8.42 a	287.77 ± 3.74 b
Organic 24 h	9.41 ± 0.36 c	15.10 ± 0.01 a	30.61 ± 0.73 a	134.36 ± 0.11 b	119.38 ± 1.52 d
Organic 48 h	4.52 ± 0.12 d	15.10 ± 0.01 a	22.76 ± 0.18 c	94.11 ± 3.67 d	51.08 ± 1.7 g
P > F (Model)	<0.0001	0.006	0.002	<0.0001	<0.0001
Significant	Yes	Yes	Yes	Yes	Yes

^1^ The ANOVA *p*-value is used to show the data as the mean ± SE (standard error). ^2^ With the 5% level of probability (*p* < 0.05), the means in the column that are followed by the same letter do not differ substantially (n.s.). Variations in color intensity reflect differences in compound concentrations across cultivation systems and fermentation durations.

**Table 2:** The concentrations of sugars in fireweed (*Chamerion angustifolium* (L.) Holub) leaves (in mg 100 g^−1^ D.M.).

Category	Total Content of Sugar	Sucrose	Glucose	Fructose
Natural 0 h	7.36 ± 0.04 ^1^ a ^2^	0.69 ± 0.036 c	4.12 ± 0.005 a	2.55 ± 0.003 a
Natural 24 h	6.74 ± 0.01 b	0.92 ± 0.006 b	3.39 ± 0.003 b	2.43 ± 0.009 a
Natural 48 h	6.46 ± 0.01 b	1.28 ± 0.005 a	2.94 ± 0.005 c	2.24 ± 0.001 ab
Biodynamic 0 h	6.63 ± 0.03 b	0.44 ± 0.012 d	4.19 ± 0.004 a	2.00 ± 0.011 b
Biodynamic 24 h	6.72 ± 0.04 b	0.61 ± 0.005 c	3.83 ± 0.001 b	2.27 ± 0.033 ab
Biodynamic 48 h	6.29 ± 0.01 b	0.68 ± 0.002 c	3.52 ± 0.003 b	2.09 ± 0.001 b
Organic 0 h	6.25 ± 0.01 b	0.69 ± 0.009 c	3.72 ± 0.005 b	1.84 ± 0.005 c
Organic 24 h	6.22 ± 0.01 b	1.21 ± 0.002 a	3.09 ± 0.010 b	1.93 ± 0.006 c
Organic 48 h	6.07 ± 0.01 b	1.01 ± 0.003 ab	3.20 ± 0.007 b	1.86 ± 0.003 c
P > F (Model)	<0.0001	<0.0001	<0.0001	<0.0001
Significant	Yes	Yes	Yes	Yes

^1^ The ANOVA *p*-value is used to show the data as the mean ± SE (standard error). ^2^ With the 5% level of probability (*p* < 0.05), the means in the column that are followed by the same letter do not differ substantially (n.s.). Variations in color intensity reflect differences in compound concentrations across cultivation systems and fermentation durations.

**Table 3:** The vitamin C content in the fireweed (*Chamerion angustifolium* (L.) Holub) leaves (in mg 100 g^−1^ D.M.).

Category	Vitamin C	Dehydroascorbic Acid	L-Ascorbic Acid
Natural 0 h	183.62 ± 0.67 ^1^ c ^2^	50.28 ± 0.42 a	133.34 ± 1.00 cd
Natural 24 h	196.74 ± 2.51 bc	52.88 ± 2.55 a	143.86 ± 0.10 c
Natural 48 h	225.25 ± 0.55 a	65.58 ± 0.36 a	159.68 ± 0.21 b
Biodynamic 0 h	190.69 ± 7.10 bc	56.37 ± 7.16 a	134.32 ± 0.40 cd
Biodynamic 24 h	208.89 ± 0.42 b	50.26 ± 0.55 a	158.63 ± 0.14 b
Biodynamic 48 h	193.32 ± 4.97 bc	59.47 ± 5.06 a	133.85 ± 0.16 cd
Organic 0 h	210.22 ± 3.58 a	58.84 ± 1.27 a	151.39 ± 3.28 b
Organic 24 h	236.24 ± 6.38 a	60.47 ± 6.65 a	175.77 ± 0.29 a
Organic 48 h	190.24 ± 0.60 b	73.79 ± 0.41 a	116.45 ± 0.33 d
P > F (Model)	<0.0001	0.042	<0.0001
Significant	Yes	Yes	Yes

^1^ The ANOVA *p*-value is used to show the data as the mean ± SE (standard error). ^2^ With the 5% level of probability (*p* < 0.05), the means in the column that are followed by the same letter do not differ substantially (n.s.). Variations in color intensity reflect differences in compound concentrations across cultivation systems and fermentation durations.

**Table 4:** Pearson’s correlation coefficients between fermentation treatments and vitamin C contents in fireweed (*Chamerion angustifolium* (L.) Holub) leaves.

	B-24 h	B-48 h	B-0 h	N-24 h	N-48 h	N-0	O-24 h	O-48 h	O-0 h	Zeaxanthin	Lutein	ß-Carotene	Chlorophyll a	Chlorophyll b	−1.000
B-24 h ^1^	**1.000**	−0.130	−0.130	−0.104	−0.130	−0.130	−0.130	−0.130	−0.130	−0.072	0.010	0.488	−0.229	−0.020	−0.800
B-48 h	−0.130	**1.000**	−0.130	−0.104	−0.130	−0.130	−0.130	−0.130	−0.130	−0.003	0.543	0.314	−0.304	−0.255	−0.500
B-0 h	−0.130	−0.130	**1.000**	−0.104	−0.130	−0.130	−0.130	−0.130	−0.130	−0.451	0.218	0.535	0.164	0.410	−0.300
N-24 h	−0.104	−0.104	−0.104	**1.000**	−0.104	−0.104	−0.104	−0.104	−0.104	−0.214	0.081	−0.347	−0.062	−0.082	0.000
N-48 h	−0.130	−0.130	−0.130	−0.104	**1.000**	−0.130	−0.130	−0.130	−0.130	0.364	0.136	−0.405	−0.252	−0.368	0.300
N-0 h	−0.130	−0.130	−0.130	−0.104	−0.130	**1.000**	−0.130	−0.130	−0.130	0.586	−0.138	−0.103	0.716	0.598	0.500
O-24 h	−0.130	−0.130	−0.130	−0.104	−0.130	−0.130	**1.000**	−0.130	−0.130	−0.094	0.067	−0.091	−0.134	−0.119	0.800
O-48 h	−0.130	−0.130	−0.130	−0.104	−0.130	−0.130	−0.130	**1.000**	−0.130	−0.103	−0.527	−0.434	−0.376	−0.493	1.000
O-0 h	−0.130	−0.130	−0.130	−0.104	−0.130	−0.130	−0.130	−0.130	**1.000**	−0.047	−0.376	−0.015	0.465	0.314	
Zeaxanthin	−0.072	−0.003	−0.451	−0.214	0.364	0.586	−0.094	−0.103	−0.047	**1.000**	−0.183	−0.290	0.290	0.104	
Lutein	0.010	0.543	0.218	0.081	0.136	−0.138	0.067	−0.527	−0.376	−0.183	**1.000**	0.389	−0.225	0.016	
ß-carotene	0.488	0.314	0.535	−0.347	−0.405	−0.103	−0.091	−0.434	−0.015	−0.290	0.389	**1.000**	0.090	0.412	
chlorophyll a	−0.229	−0.304	0.164	−0.062	−0.252	0.716	−0.134	−0.376	0.465	0.290	−0.225	0.090	**1.000**	0.911	
chlorophyll b	−0.020	−0.255	0.410	−0.082	−0.368	0.598	−0.119	−0.493	0.314	0.104	0.016	0.412	0.911	**1.000**	

^1^ B—biodynamic cultivation; O—organic cultivation; N—natural cultivation. 0 h (Control)—non-fermented leaves; 24 h and 48 h—fermentation duration. Values represent Pearson’s correlation coefficients (r) between treatments and pigment contents. Positive correlations are shown in red, and negative correlations in blue. The strength of correlation increases with color intensity. Statistical significance was assessed by ANOVA (*p* < 0.05).

**Table 5:** Pearson’s correlation coefficients between fermentation treatments and sugar contents in fireweed (*Chamerion angustifolium* (L.) Holub) leaves.

	B-0 h	B-24 h	B-48 h	N-0 h	N-24 h	N-48 h	O-0 h	O-24 h	O-48 h	Sucrose	Glucose	Fructose	Total Content of Sugar	−1.000
B-0 h ^1^	**1.000**	−0.130	−0.130	−0.104	−0.130	−0.130	−0.130	−0.130	−0.130	−0.529	0.576	−0.190	0.146	−0.800
B-24 h	−0.130	**1.000**	−0.130	−0.104	−0.130	−0.130	−0.130	−0.130	−0.130	−0.303	0.260	0.247	0.241	−0.500
B-48 h	−0.130	−0.130	**1.000**	−0.104	−0.130	−0.130	−0.130	−0.130	−0.130	−0.215	−0.015	−0.045	−0.222	−0.300
N-0 h	−0.104	−0.104	−0.104	**1.000**	−0.104	−0.104	−0.104	−0.104	−0.104	−0.173	0.413	0.545	0.734	0.000
N-24 h	−0.130	−0.130	−0.130	−0.104	**1.000**	−0.130	−0.130	−0.130	−0.130	0.104	−0.124	0.484	0.262	0.300
N-48 h	−0.130	−0.130	−0.130	−0.104	−0.130	**1.000**	−0.130	−0.130	−0.130	0.578	−0.521	0.188	−0.041	0.500
O-0 h	−0.130	−0.130	−0.130	−0.104	−0.130	−0.130	**1.000**	−0.130	−0.130	−0.202	0.164	−0.433	−0.258	0.800
O-24 h	−0.130	−0.130	−0.130	−0.104	−0.130	−0.130	−0.130	**1.000**	−0.130	0.487	−0.392	−0.299	−0.289	1.000
O-48 h	−0.130	−0.130	−0.130	−0.104	−0.130	−0.130	−0.130	−0.130	**1.000**	0.223	−0.293	−0.406	−0.454	
Sucrose	−0.529	−0.303	−0.215	−0.173	0.104	0.578	−0.202	0.487	0.223	**1.000**	−0.936	−0.046	−0.367	
Glucose	0.576	0.260	−0.015	0.413	−0.124	−0.521	0.164	−0.392	−0.293	−0.936	**1.000**	0.175	0.584	
Fructose	−0.190	0.247	−0.045	0.545	0.484	0.188	−0.433	−0.299	−0.406	−0.046	0.175	**1.000**	0.857	
Total content of sugar	0.146	0.241	−0.222	0.734	0.262	−0.041	−0.258	−0.289	−0.454	−0.367	0.584	0.857	**1.000**	

^1^ B—biodynamic cultivation; O—organic cultivation; N—natural cultivation. 0 h (Control)—non-fermented leaves; 24 h and 48 h—fermentation duration. Values represent Pearson’s correlation coefficients (r) between treatments and pigment contents. Positive correlations are shown in red, and negative correlations in blue. The strength of correlation increases with color intensity. Statistical significance was assessed by ANOVA (*p* < 0.05).

**Table 6:** Pearson’s correlation coefficients between fermentation treatments and vitamin C contents in fireweed (*Chamerion angustifolium* (L.) Holub) leaves.

	B-0 h	B-24 h	B-48 h	N-0 h	N-24 h	N-48 h	O-0 h	O-24 h	O-48 h	Vitamin C	Dehydroascorbic Acid	L-Ascorbic Acid	−1.000
B-0 h	**1.000**	−0.130	−0.104	−0.130	−0.130	−0.130	−0.130	−0.130	−0.130	−0.264	−0.074	−0.239	−0.800
B-24 h	−0.130	**1.000**	−0.104	−0.130	−0.130	−0.130	−0.130	−0.130	−0.130	0.095	−0.299	0.271	−0.500
B-48 h	−0.104	−0.104	**1.000**	−0.104	−0.104	−0.104	−0.104	−0.104	−0.104	−0.219	−0.065	−0.196	−0.300
N-0 h	−0.130	−0.130	−0.104	**1.000**	−0.130	−0.130	−0.130	−0.130	−0.130	−0.404	−0.298	−0.260	0.000
N-24 h	−0.130	−0.130	−0.104	−0.130	**1.000**	−0.130	−0.130	−0.130	−0.130	−0.145	−0.203	−0.039	0.300
N-48 h	−0.130	−0.130	−0.104	−0.130	−0.130	**1.000**	−0.130	−0.130	−0.130	0.418	0.266	0.293	0.500
O-0 h	−0.130	−0.130	−0.104	−0.130	−0.130	−0.130	**1.000**	−0.130	−0.130	0.121	0.017	0.119	0.800
O-24 h	−0.130	−0.130	−0.104	−0.130	−0.130	−0.130	−0.130	**1.000**	−0.130	0.634	0.077	0.631	1.000
O-48 h	−0.130	−0.130	−0.104	−0.130	−0.130	−0.130	−0.130	−0.130	**1.000**	−0.273	0.568	−0.614	
Vitamin C	−0.264	0.095	−0.219	−0.404	−0.145	0.418	0.121	0.634	−0.273	**1.000**	0.376	0.850	
Dehydroascorbic acid	−0.074	−0.299	−0.065	−0.298	−0.203	0.266	0.017	0.077	0.568	0.376	**1.000**	−0.169	
L-ascorbic acid	−0.239	0.271	−0.196	−0.260	−0.039	0.293	0.119	0.631	−0.614	0.850	−0.169	**1.000**	

^1^ B—biodynamic cultivation; O—organic cultivation; N—natural cultivation. 0 h (Control)—non-fermented leaves; 24 h and 48 h—fermentation duration. Values represent Pearson’s correlation coefficients (r) between treatments and pigment contents. Positive correlations are shown in red, and negative correlations in blue. The strength of correlation increases with color intensity. Statistical significance was assessed by ANOVA (*p* < 0.05).

## Data Availability

The original contributions presented in this study are included in the article. Further inquiries can be directed to the corresponding author.

## Abbreviations

The following abbreviations are used in this manuscript:

SSF	Solid-state fermentation
UV	ultraviolet
0 h	0 hours
24 h	24 hours
48 h	48 hours
B	biodynamic cultivation
O	organic cultivation
N	natural cultivation
